# Comparative bibliometric insights into Chinese and international research on *Suaeda* over 20 years

**DOI:** 10.3389/fpls.2026.1866245

**Published:** 2026-06-26

**Authors:** Yihao Cui, Shuang Liu, Su Xie, Zhizhong Shen

**Affiliations:** 1 College of Humanities & Social Development, Nanjing Agricultural University, Nanjing, China; 2 School of Economics and Management, Wuchang Shouyi University, Wuhan, China; 3 Research Center for Hubei Agricultural Modernization and Rural Development, Wuhan, China

**Keywords:** bibliometrics, CiteSpace, development trend, research hotspots, *Suaeda*

## Abstract

**Introduction:**

*Suaeda* is a halophytic genus widely distributed in coastal salt marshes and inland saline-alkaline habitats, and it has attracted increasing attention because of its ecological importance and resource utilization potential. Among its members, *S.glauca* is one of the representative and most extensively studied species in China. In recent years, research on *Suaeda* has expanded steadily, yet existing studies are mostly confined to specific topics and still lack a systematic comparison between Chinese-language and international English-language scholarship.

**Methods:**

To address this gap, this study applies CiteSpace-based bibliometric methods to comparatively analyze *Suaeda*-related literature in the China National Knowledge Infrastructure and the Web of Science Core Collection from 2005 to 2025. Publication trends, country and institutional collaboration networks, keyword co-occurrence, and temporal evolution were examined to reveal the knowledge structure, research hotspots, and developmental trajectories of the field.

**Results:**

The annual number of publications on *Suaeda* has generally increased over the past two decades, although periodic fluctuations remain evident. China ranks first in both publication output and betweenness centrality, indicating its prominent position in this research area. In terms of thematic focus, Chinese-language studies place greater emphasis on the practical application of *Suaeda* in saline-alkaline land improvement and ecological restoration, whereas international English-language studies pay more attention to physiological stress-tolerance mechanisms and ecosystem-level functions.

**Discussion:**

Chinese-language research has gone through a development path from habitat investigation and resource census, to ecological restoration and saline-alkali land management, and then to more diversified functional and technological applications; and international research starts from ecological responses and population performance, gradually delving into physiological mechanisms, rhizosphere processes and multi-scale regulation. The cutting-edge hotspots in recent years include germplasm resource evaluation, remote sensing applications, plant-soil feedback, cell wall processes, and rhizosphere mediated ecological interactions, etc. Species nomenclatural confusion has caused inconsistent and unrepeatable results in salt tolerance physiology and restoration practices. Future research should strengthen taxonomic accuracy, deepen the integration of applied studies and mechanistic investigations, and promote broader international collaboration. This study provides a foundation for integrating applied saline-alkali land restoration with mechanistic stress physiology research.

## 1 Introduction

*Suaeda*, belonging to the family Amaranthaceae and the genus *Suaeda Forsk. ex Scop.*, is a group of annual or perennial succulent halophytes widely distributed in coastal and inland saline habitats. These plants play an important role in maintaining soil stability and improving the ecological environment (Huang et al., 2025). As typical euhalophytes, *Suaeda* species are naturally found in extreme environments such as seacoasts, saline-alkaline wastelands, canal banks, field margins, and low-lying inland lake basins (Lu et al., 2024). Most species in this genus lack specialized salt-secreting structures and instead rely on marked leaf succulence, ion compartmentalization, osmotic adjustment, and tissue dilution to cope with salt stress. These physiological strategies confer strong tolerance to salinity, waterlogging, and nutrient-poor conditions. Among them, *Suaeda glauca* is one of the representative and relatively well-studied taxa in China, with a salt tolerance capacity of approximately 25 g·kg^-1^ and an upper tolerance limit near 35 g·kg^-1^ (Zhang et al., 2007).

China is one of the major distribution and utilization centers of *Suaeda*. As early as the Ming Dynasty, the 《Jiuhuang Bencao》 published in 1406 and attributed to Zhu Su systematically recorded the morphology, flavor properties, and edible uses of *Suaeda*, highlighting its long-standing role in subsistence and traditional plant use (Zhao and Zhang, 2015; Zhu., 2008). The genus *Suaeda* is highly diverse in China, with 20 species and one variety recorded across the northeastern, northern, northwestern, and eastern coastal regions, including Heilongjiang, Inner Mongolia, Hebei, Shandong, Jiangsu, Henan, Ningxia, and Qinghai (Sun et al., 2005). Among these taxa, *S. glauca* and *S. salsa* are common dominant species in coastal wetlands and inland saline-alkaline habitats, and in some regions they contribute to the formation of characteristic red beach landscapes (Cui et al., 2019; Lu et al., 2024). As a characteristic plant group of saline-alkaline ecosystems, *Suaeda* has attracted considerable attention for its ecological functions and adaptive strategies. Through succulent dilution, ion compartmentalization, and related salt-tolerance mechanisms, *Suaeda* species can accumulate salts in their tissues while maintaining growth under harsh environmental conditions. At the same time, they contribute to the amelioration of saline-alkaline soils by improving soil physicochemical properties, including reductions in bulk density, increases in organic matter content, and promotion of aggregate formation (Ni et al., 2025; Wang et al., 2025a, b; Zhang et al., 2025a). Previous studies have also shown that *Suaeda* vegetation can enhance the abundance and diversity of soil microbial communities (Tang et al., 2023; Yue et al., 2023). In addition, *Suaeda* species show considerable potential in the remediation of soils contaminated by heavy metals and organic pollutants (Xu et al., 2025a; Zhang et al., 2025b), as well as in the treatment of eutrophic water bodies (Chen et al., 2017). These features make the genus valuable not only for ecological restoration but also for landscape enhancement in saline-alkali environments (Song et al., 2022).

From the perspective of resource utilization, *Suaeda* also holds broad developmental potential. Its tender shoots can be consumed as vegetables and are rich in proteins, carotenoids, and mineral nutrients. The seeds contain abundant unsaturated fatty acids, especially linoleic acid, and therefore have potential for the production of edible oil and functional foods. Plant residues can be incorporated into soil as green manure, and the straw can also be developed as a feed additive (Hong et al., 2023; Liu et al., 2022; Xu et al., 2025b; Yu et al., 2011). This resource value is especially relevant in the broader context of global soil salinization. According to the Food and Agriculture Organization, saline-alkali soils occupy approximately 1.38 billion hectares worldwide and continue to expand by 1 to 2 million hectares annually (Liu et al., 2026). The resulting economic losses are estimated to exceed 27 billion USD each year (Zörb et al., 2019). In China alone, about 2 million hectares of saline-alkaline wasteland are considered suitable for *Suaeda* cultivation. Based on a seed yield of 1500 kg per hectare, the annual seed production of *Suaeda* could reach about 3 million tons (Ma et al., 2021). These figures indicate substantial opportunities for the ecological and industrial development of *Suaeda* in saline-alkaline land improvement, marginal land utilization, bio-energy development, and functional food innovation.

Bibliometrics is a literature-based analytical approach grounded in mathematical statistics and information mining. It enables both quantitative and qualitative evaluation of research outputs from multiple perspectives, including institutions, keywords, citation relationships, and countries of origin (Chen et al., 2024; Liu et al., 2025). By doing so, it helps reveal publication trends, knowledge structures, collaboration patterns, and shifts in research hotspots within a given field. In recent years, bibliometric methods have been widely applied in botany, ecology, and environmental science. Although some bibliometric studies on *Suaeda* have already been conducted, most have focused primarily on domestic research or on limited thematic scopes, and systematic comparison between Chinese-language and international English-language literature remains insufficient (Ma et al., 2021). This gap restricts a comprehensive understanding of global collaboration networks, core research groups, thematic evolution, and differences in research priorities and paradigms between Chinese and international studies on *Suaeda*.

Against this background, the present study integrates literature data from the China National Knowledge Infrastructure (CNKI) and the Web of Science (WoS) Core Collection to conduct a systematic bibliometric analysis of *Suaeda*-related research using CiteSpace. Particular attention is given to representative species such as *S. glauca* where relevant. The objectives are to clarify the development history and publication trends of *Suaeda* research in China and abroad, identify major research entities including countries and institutions and map their collaborative networks, and compare hotspot topics and frontier evolution patterns in Chinese and international studies. By doing so, this study aims to provide a clearer knowledge map of the field and to offer scientific support for future research planning, interdisciplinary integration, and international collaboration in the ecological restoration and high-value utilization of *Suaeda* in saline-alkaline environments.

## 2 Materials and methods

### 2.1 Data source and data sorting

Chinese-language literature was retrieved from the CNKI database, and English-language literature was collected from the WoS Core Collection database. To ensure that the research scope is strictly limited to the natural growth conditions of *Suaeda* in the field environment (especially in saline-alkali land), the retrieval strategy was designed around *Suaeda*-related terms combined with the keyword “soil”. In the CNKI database, the topic search included “碱蓬” and related *Suaeda* terms together with “土壤”, and the document type was limited to academic journal articles published between January 1, 2005 and December 31, 2025. Because the term “碱蓬” is sometimes used broadly or ambiguously in Chinese literature, manual screening was conducted after retrieval to retain studies genuinely related to *Suaeda*. The keyword “soil” was included to exclude studies focused mainly on hydroponics or substrate cultivation and to ensure that the selected literature was relevant to soil environments and saline-alkaline land contexts. A total of 950 Chinese records were initially obtained. For the WoS Core Collection, the search was conducted using *Suaeda*-related terms in combination with “soil” over the same time span. This search yielded 257 English records.

After manual screening of both datasets to remove duplicate records, irrelevant publications, conference papers, and review articles. All information, including the number of papers and citations, title, author, affiliation, country, keywords, journal, publication year, and references, were collected. Finally, a total of 939 Chinese publications and 210 English publications were retained for subsequent bibliometric analysis.

### 2.2 Data analysis

**Figure 1:**
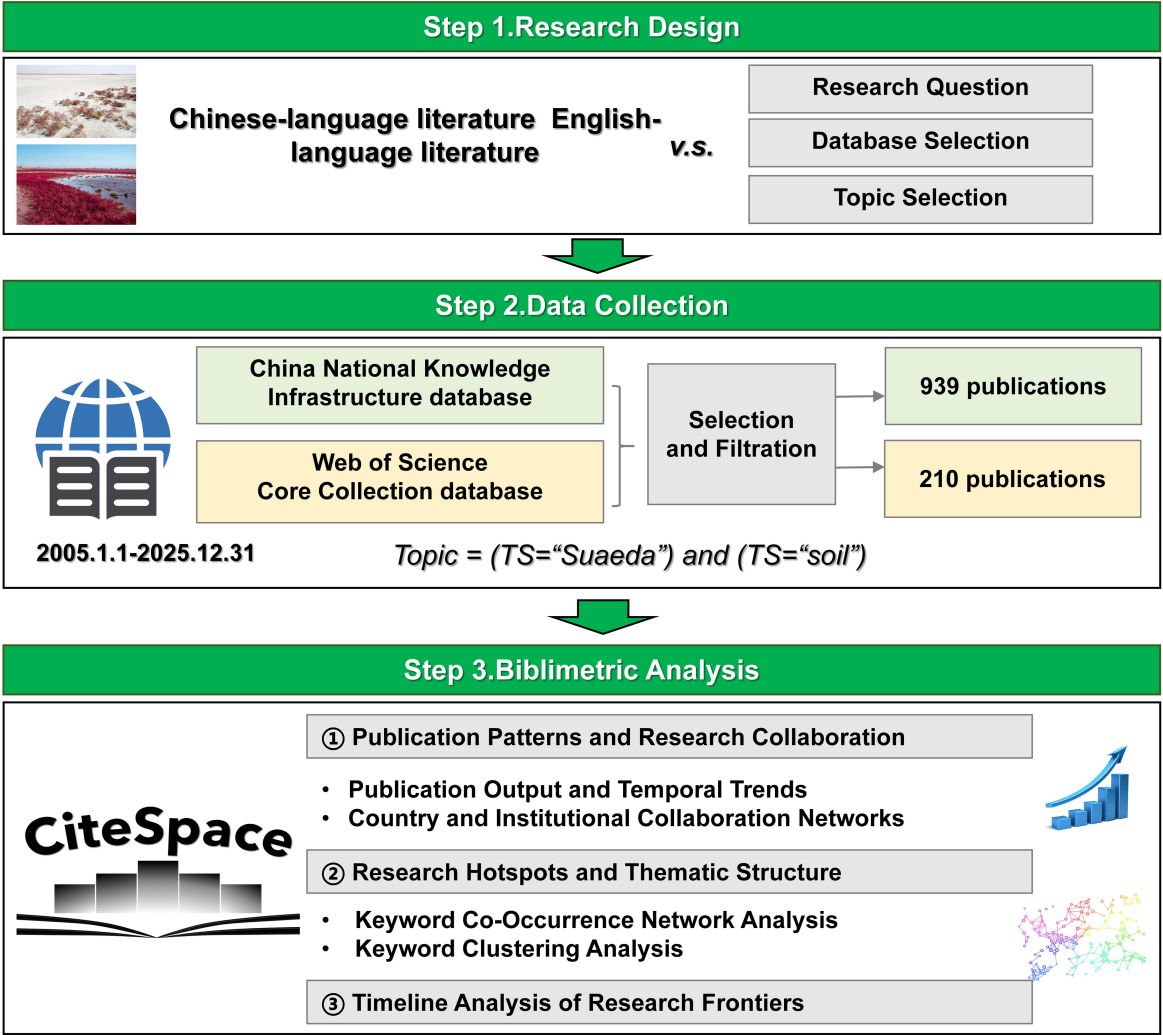
Flowchart of CiteSpace bibliometric analysis.

CiteSpace is a widely used bibliometric visualization tool for mapping knowledge structures, collaboration networks, and research trends in scientific literature (Geng et al., 2024; Yang and Liu, 2024). In this study, CiteSpace 6.4.R1 was used to analyze the retained records from CNKI and the WoS Core Collection databases. The software was applied to examine annual publication trends, major publication outlets, country and institutional distributions, keyword co-occurrence patterns, and temporal evolution in soil-related research on *Suaeda* (**Figure 1**). Through co-occurrence networks and clustering analysis, CiteSpace helped reveal the major research hotspots, thematic structure, and developmental trajectory of this field (Xu et al., 2023; Yang et al., 2025; Zhu et al., 2025). The time span for both Chinese and English literature was set from 2005 to 2025, with time slices set to 1 year. The selection criteria were based on the g-index (k = 25), with LRF = 2.5, L/N = 10, and e = 1.0. All other settings were kept as system defaults. Microsoft Excel 2021 was used to organize the bibliographic data and generate figures for annual publication trends and related descriptive statistics. The combined use of CiteSpace and Excel enabled both visual and quantitative comparison of Chinese and international studies on *Suaeda*.

## 3 Results

### 3.1 Publication output and temporal trends

**Figure 2:**
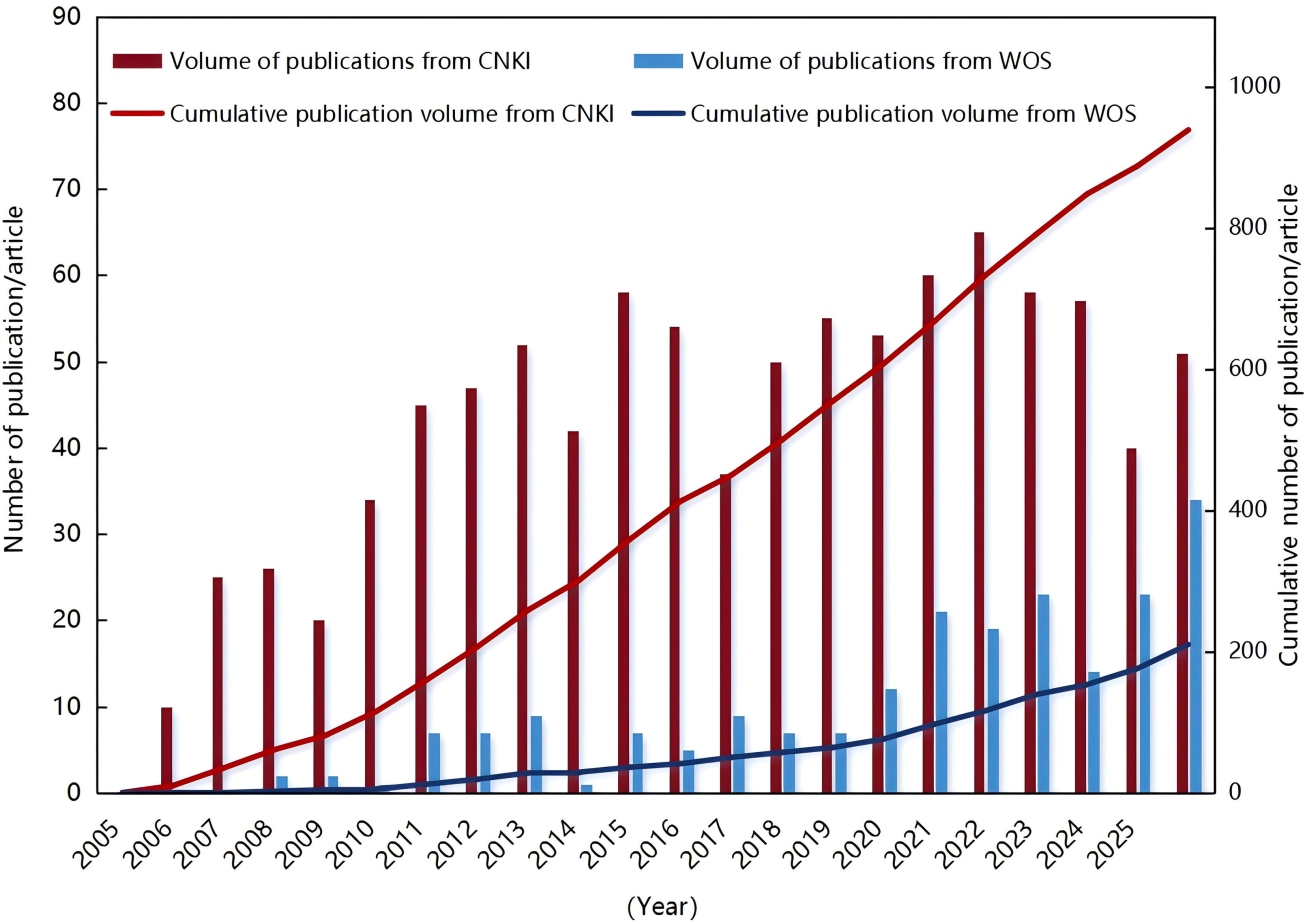
Annual and cumulative numbers of publications on *Suaeda*-related research in the CNKI and WoS Core Collection databases from 2005 to 2025.

As illustrated in **Figure 2**, the annual publication output of *Suaeda*-related studies in both the CNKI and WoS core collection databases generally exhibited a fluctuating upward trend from 2005 to 2025. However, the two databases differed notably in the timing, magnitude, and pace of growth. Publications indexed in the CNKI appeared earlier and increased more rapidly overall, reaching a peak of 65 papers in 2021. This pattern suggests that research on *Suaeda* in China developed at an earlier stage and has long maintained relatively high scholarly attention. In contrast, the WoS Core Collection dataset showed a steadier but later increase, with the highest annual output recorded in 2025 (34 papers). Compared with the CNKI dataset, the international rise of *Suaeda* research appears to have occurred more recently. It suggests that *Suaeda* research has gradually expanded from a regionally concentrated applied topic into a broader field of international interest.

### 3.2 Country and institutional collaboration networks

**Figure 3:**
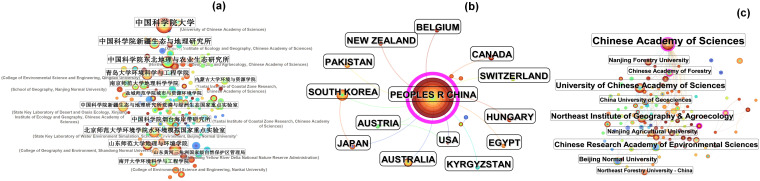
Institutional collaboration network in the CNKI database **(A)**, and country-level **(B)** and institutional **(C)** collaboration networks in the WoS Core Collection database for *Suaeda*-related research. Node size represents publication frequency, and node color reflects the temporal distribution of publications. Purple outer rings indicate higher betweenness centrality.

**Figure 3** shows the country-level and institutional collaboration networks of *Suaeda*-related studies over the past two decades. At the country level, China ranked first in publication output, with 144 papers in the WoS dataset, indicating its leading role in this field. China also showed the highest betweenness centrality (1.12), whereas the centrality values of other countries were close to 0. This result indicates that China occupies the most important bridging position in the international collaboration network of *Suaeda* research and plays a central role in connecting different research groups and knowledge clusters.

At the institutional level, the major contributors in both the CNKI and WoS Core Collection databases were dominated by Chinese universities and research institutes (**Figures 3A, C**), further highlighting the prominent position of China in this field. Among them, the Chinese Academy of Sciences and its affiliated institutes were the most influential contributors. In the WoS Core Collection database, the Chinese Academy of Sciences ranked first in publication output and also exhibited relatively high centrality, indicating its dual role as a major research producer and an important collaborative hub. In the CNKI database, high-output institutions included the University of Chinese Academy of Sciences, the Xinjiang Institute of Ecology and Geography, the Northeast Institute of Geography and Agroecology, and several universities located in ecologically sensitive coastal regions. At the same time, institutions in inland saline-alkali regions such as Xinjiang and Inner Mongolia also made substantial contributions, indicating that the research scope of *Suaeda* has expanded from coastal wetland systems to inland saline-alkali ecosystems.

Betweenness centrality values also reveal differences between publication productivity and collaborative influence (**Table 1**). A small number of institutions, represented by the Chinese Academy of Sciences, had relatively high centrality values in both databases, indicating that they maintained broader and closer collaborative links across institutions and disciplines. By contrast, some institutions with relatively high publication output showed limited centrality, suggesting that their research activities were more locally concentrated or less integrated into wider collaboration networks. In other words, high productivity does not necessarily correspond to a strong coordinating role in the knowledge network.

**Table 1:** Top 10 institutions by publication output in *Suaeda*-related research in the CNKI and WoS core collection databases.

Rank	CNKI	Frequency	Centrality	Year	WOS Core collection	Frequency	Centrality	Year
1	University of Chinese Academy of Sciences	38	0.10	2005	Chinese Academy of Sciences	39	0.59	2009
2	Xinjiang Institute of Ecology and Geography, Chinese Academy of Sciences	24	0.02	2007	University of Chinese Academy of Sciences	15	0.04	2009
3	Northeast Institute of Geography and Agroecology, Chinese Academy of Sciences	22	0.04	2007	Northeast Institute of Geography & Agroecology	14	0.04	2010
4	College of Environmental Science and Engineering, Qingdao University	14	0.02	2016	Northwest A&F University - China	13	0.09	2010
5	State Key Laboratory of Water Environment Simulation, School of Environment, Beijing Normal University	14	0.01	2009	Agriculture & Agri Food Canada	8	0.05	2010
6	Yantai Institute of Coastal Zone Research, Chinese Academy of Sciences	12	0.04	2010	Chinese Academy of Agricultural Sciences	7	0.09	2017
7	College of Geography and Environment, Shandong Normal University	12	0.03	2016	University of Western Australia	7	0.05	2016
8	School of Geography, Nanjing Normal University	10	0.01	2011	China Agricultural University	6	0.12	2010
9	College of Environmental Science and Engineering, Nankai University	8	0.01	2008	Institute of Soil & Water Conservation (ISWC)	6	0.05	2010
10	Shandong Yellow River Delta National Nature Reserve Administration	7	0.02	2008	Gansu Agricultural University	5	0.03	2021

### 3.3 Keyword co-occurrence network analysis

**Figure 4:**
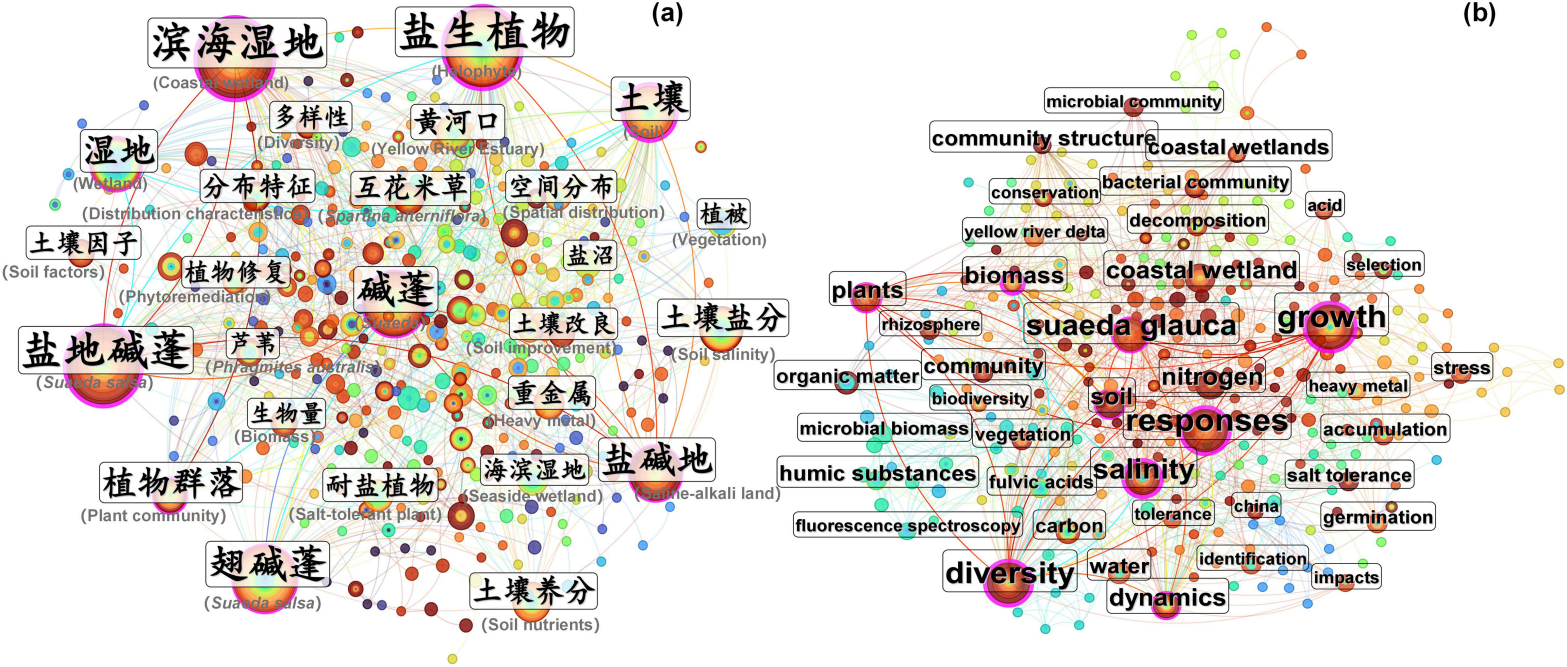
Co-occurrence knowledge graph and frequency statistics of core keywords from the database of CNKI **(A)** and WOS Core Collection **(B)**. Node size is proportional to keyword co-occurrence frequency. Purple outer rings indicate nodes with a betweenness centrality ≥ 0.1, signifying their important bridging role in the network.

Keyword co-occurrence analysis is useful for identifying major research hotspots and comparing thematic preferences across different literature datasets (Liu et al., 2025). As shown in **Figure 4**, the keyword network of the CNKI database consists of 456 nodes and 974 links, with a network density of 0.0094, whereas the WoS Core Collection database contains 343 nodes and 1239 links, with a higher network density of 0.0211. This indicates that the WoS Core Collection keyword network is more tightly connected and that international studies tend to show relatively stronger thematic associations.

The temporal distribution of keyword emergence also reveals clear differences between the two databases (**Table 2**). In the CNKI dataset, most high-frequency keywords first appeared between 2005 and 2007, indicating that *Suaeda*-related research in China entered a relatively active stage at an earlier time. By contrast, most core keywords in the WoS Core Collection dataset emerged after 2010, with some appearing as late as 2017, suggesting that international attention to *Suaeda* developed later. Taken together, these patterns imply that early accumulation in this field was concentrated more strongly in Chinese literature, whereas the international literature expanded more visibly in the later period.

**Table 2:** List of TOP 10 keywords from the databases of CNKI and WOS core collection.

Rank	CNKI	Frequency	Centrality	Year	WOS	Frequency	Centrality	Year
1	Halophytes	87	0.28	2006	Growth	23	0.28	2011
2	Coastal Wetlands	83	0.30	2010	Responses	22	0.27	2009
3	*Suaeda salsa*	80	0.26	2005	Diversity	21	0.26	2010
4	*Suaeda glauca*	55	0.18	2007	*Suaeda glauca*	17	0.20	2017
5	*Suaeda heteroptera*	54	0.16	2006	Salinity	16	0.22	2009
6	Saline-alkali soil	44	0.10	2005	Dynamics	10	0.17	2012
7	Soil	42	0.23	2006	Nitrogen	10	0.05	2017
8	Wetlands	39	0.15	2006	Coastal wetland	10	0.04	2017
9	Plant communities	31	0.12	2007	Biomass	9	0.24	2010
10	Soil salinity	23	0.08	2006	Plants	9	0.10	2019

In terms of thematic content, the two datasets show different research orientations. In the CNKI database, high-frequency keywords such as “coastal wetlands (滨海湿地)”, “halophytes (盐生植物)”, “soil (土壤)” indicate a strong emphasis on ecological restoration, saline-alkali land improvement, wetland vegetation, and field-based applications. These keywords reflect the practical orientation of domestic research, which has long been closely linked to coastal wetland management, vegetation reconstruction, and the utilization of salt-tolerant plants under regional ecological conditions. In addition, multiple species-related terms, including *S. salsa*, *S. glauca*, and *S. heteroptera*, appeared frequently in the CNKI dataset, suggesting that domestic studies often address *Suaeda* at both genus and species levels. A notable feature of the CNKI dataset is the coexistence of different Chinese common names and scientific names for *Suaeda* species. This pattern suggests a certain degree of nomenclatural heterogeneity in the literature, especially in the use of terms such as “碱蓬”, *S. glauca*, and *S. salsa*.

In contrast, the WoS Core Collection database shows a stronger emphasis on physiological and ecological mechanisms. High-frequency keywords such as “growth”, “responses”, “salinity”, “dynamics”, “nitrogen”, and “biomass” indicate that international studies are more concerned with plant responses to environmental stress, nutrient cycling, and ecological processes. This tendency is also reflected in representative studies within the WoS literature. For example, some studies have explored the relationship between *Suaeda* communities and soil dissolved organic matter or carbon and nitrogen dynamics (Yu et al., 2010), while others have examined salt-responsive transcriptomic regulation in *S. glauca* (Rodriguez-Uribe et al., 2011) or the beneficial effects of *Suaeda*-associated rhizosphere microorganisms on crop salt resistance (Wang et al., 2022). These studies collectively show that international research has paid increasing attention to the mechanistic basis of stress tolerance, plant–soil interactions, and the ecological functions of *Suaeda* in saline-alkali environments.

### 3.4 Keyword clustering analysis

As shown in **Figure 4**, the keyword clustering analysis yielded Q = 0.5764 and S = 0.857 for the CNKI database, and Q = 0.66 and S = 0.8622 for the WoS Core Collection database. Since all values exceed the commonly accepted thresholds, the clustering results for both datasets can be considered reliable and suitable for further interpretation (Liu et al., 2025).

**Figure 5:**
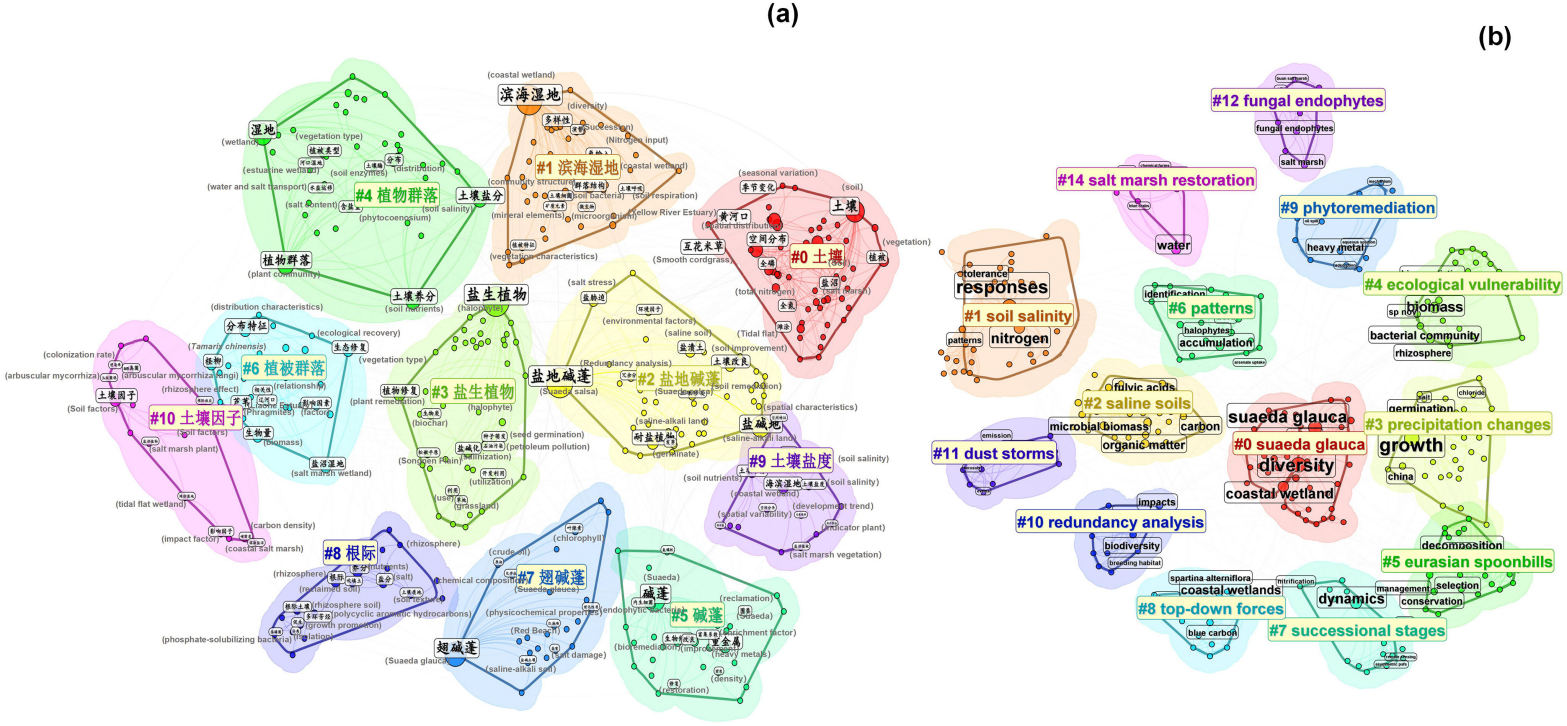
Keyword clustering map of *Suaeda*-related research in the CNKI [**(a)**, Modularity Q = 0.5764, Weighted Mean Silhouette S = 0.857] and WOS Core Collection [**(b)**, Modularity Q = 0.66, Weighted Mean Silhouette S = 0.8622].

Keyword clustering analysis helps reveal the internal structure, major themes, and developmental trends of a research field. Combined with **Figure 5** and **Table 3**, the clustering results show clear thematic differences between the two datasets. In the CNKI database, the major clusters include “soil (土壤)”, “coastal wetland (滨海湿地)”, “*Suaeda salsa* (盐地碱蓬)”, “halophytes (盐生植物)”, “plant community (植物群落)”, and “rhizosphere (根际)”, indicating that domestic studies mainly focus on soil environments, wetland ecosystems, species utilization, and vegetation restoration. In the WoS Core Collection database, representative clusters include “*Suaeda glauca*”, “soil salinity”, “precipitation change”, “ecological vulnerability”, “top-down forces”, “fungal endophytes”, and “salt marsh restoration”, suggesting stronger emphasis on stress responses, ecological processes, global change, and restoration mechanisms. The clustering results indicate that CNKI research is more application- and region-oriented, whereas WoS research tends to be more mechanism-driven and internationally framed.In addition, the largest cluster in the CNKI database is “#0 soil” (56 nodes; mean year 2013). Its core terms, such as “soil (土壤)”, “Yellow River Estuary (黄河口)”, “salt marsh (盐沼)”, “total nitrogen (全氮)”, and “total phosphorus (全磷)”, suggest that early domestic research mainly concentrated on the soil conditions associated with *Suaeda* habitats, particularly in typical estuarine and coastal wetlands. This cluster reflects a research pattern centered on local ecosystems, with attention to soil physicochemical properties and their effects on vegetation distribution. By contrast, the largest cluster in the WoS Core Collection database is “#0 *Suaeda glauca*” (59 nodes; mean year 2020). Its associated terms, including “exogenous gibberellin,” “dormancy release,” “microbial community”, “pore structure”, and “salt stress”, indicate that recent international studies have paid greater attention to seed ecology, physiological regulation, and plant-microbe interactions under multiple environmental stresses. Compared with the CNKI dataset, the WoS dataset therefore shows a more explicit shift toward mechanistic and fine-scale research.

**Table 3:** Comparison of keyword clustering from the databases of CNKI and WoS core collection databases.

Database	Label	Node	S value	Mean (year)	Keywords
CNKI	#0土壤 (soil)	56	0.896	2013	土壤 (soil); 黄河口 (Yellow River Estuary); 盐沼(salt marsh); 全氮 (total nitrogen); 全磷 (total phosphorus)
	#1滨海湿地 (Coastal Wetland)	50	0.768	2016	滨海湿地 (coastal wetland); 群落结构 (community structure); 植被特征 (vegetation characteristics); 多样性 (diversity); 土壤细菌 (soil bacteria)
	#2盐地碱蓬 (*Suaeda salsa*)	48	0.814	2014	盐地碱蓬 (*Suaeda salsa*); 盐碱地 (saline-alkali land); 土壤改良 (soil improvement); 盐渍土 (saline soil); 耐盐植物 (salt-tolerant plant)
	#3盐生植物 (Halophytes)	46	0.870	2013	盐生植物 (*Halophytes*); 利用 (utilization); 盐碱化(salinization); 植物修复 (phytoremediation); 石油污染 (petroleum pollution)
	#4植物群落 (plant community)	42	0.865	2011	植物群落 (plant community); 土壤盐分 (soil salinity); 土壤养分 (soil nutrients); 湿地 (wetland); 松嫩草地 (Songnen Grassland)
	#5碱蓬 (*Suaeda*)	31	0.901	2014	碱蓬 (*Suaeda*); 重金属 (heavy metal); 改良 (improvement); 围垦 (reclamation); 苜蓿 (*Alfalfa*)
	#6植被群落 (vegetation community)	31	0.820	2013	植被群落 (vegetation community); 相关性 (correlation); 分布特征 (distribution characteristics); 芦苇 (*Phragmites australis*); 辽河口 (Liaohe Estuary)
	#7翅碱蓬 (*Suaeda heteroptera*)	25	0.805	2014	翅碱蓬 (*Suaeda heteroptera*); 野外光谱 (field spectrum); 叶绿素 (Chlorophyll); 盐害 (salt injury); 红海滩 (Red Beach)
	#8根际 (rhizosphere)	19	0.915	2013	根际 (rhizosphere); 促生 (growth promotion); 盐分 (salinity); 分离 (isolation); 多环芳烃 (Polycyclic aromatic hydrocarbons)
	#9土壤盐度 (soil salinity)	16	0.948	2009	土壤盐度 (soil salinity); 海滨湿地 (coastal wetland); 盐沼植被 (salt marsh vegetation); 土壤水分 (soil moisture); 空间分异 (spatial differentiation)
	#10土壤因子 (soil factors)	16	0.975	2012	土壤因子 (soil factor); AM真菌 (AM fungi); 菌根侵染 (mycorrhizal colonization); 侵染强度 (colonization intensity); 盐沼植物 (salt marsh plant)
WoS	#0 suaeda glauca	59	0.741	2020	*suaeda glauca*; exogenous gibberellin; dormancy release; microbial community; pore structure; salt stress; cold stratification; burial depth; osmotic potential; *phragmites communis*
	#1 soil salinity	36	0.808	2017	soil salinity; vegetation pattern; environmental gradient; soil moisture; edaphic factor; *tamarix chinensis*; competitive ability; stress tolerance; functional traits; positive interaction
	#2 saline soils	34	0.885	2011	saline soil; exchangeable sodium percentage; ultraviolet-visible spectroscopy; fulvic acid; flavo-aquic soils
	#3 precipitation changes	33	0.812	2018	precipitation change; soil salinity; coastal salt marsh; dominant species; functional group; *suaeda salsa*; soil salinization; carbon utilization; soil heterogeneity; plant community composition
	#4 ecological vulnerability	26	0.942	2015	ecological vulnerability; empirical analysis; dual adaptive management; ecosystem response; landscape pattern; petroleum-contaminated soil; *suaeda glauca*; microbial community structure
	#5 eurasian spoonbills	22	0.851	2016	eurasian spoonbill; coastal wetland; habitat use; natural wetland; artificial wetland; habitat selection; variation partitioning; red-crowned crane; multiple scale
	#6 patterns	20	0.938	2018	pattern; accumulation; polybrominated diphenyl ether; south China; *suaeda glauca*; salt acclimation; sodium uptake rate; mild salinity
	#7 successional stages	17	0.879	2013	successional stages; microbial dynamics; species replacement; saline-alkali grasslands; asymmetric psfs; river estuary; seepweed wetland; biological cycle; habitat selection
	#8 top-down forces	15	0.931	2021	top-down force; sediment elevation change; red beach; bottom-up forces; salt marsh; estuarine saltmarsh; organic matter; carbon isotopes; TN ratio
	#9 phytoremediation	14	0.988	2017	phytoremediation; hyperaccumulator; heavy metal; physiological response
	#10 redundancy analysis	13	0.920	2013	redundancy analysis; soil NO_3_^--^N; vegetation succession; soil electrical conductivity; virtual population analysis; canonical correspondence analysis; plant trait; CSR strategies; coastal sand dune plants; soil factor
	#11 dust storms	11	0.968	2013	dust storms; vegetation succession; wind erosion; soil salt content
	#12 fungal endophytes	11	0.944	2016	fungal endophyte; growth promotion; coastal salt marsh plant; Buan salt marsh; genetic diversity; halophytic plant
	#14 salt marsh restoration	6	0.980	2018	*suaeda glauca*; salt marsh restoration; tidal creek; *phragmites communis*; chemical form; subcellular distribution

### 3.5 Timeline analysis of research frontiers

Keyword timeline mapping reveals not only when high-frequency terms appeared, but also how research frontiers persisted, shifted, and were replaced over time. In CiteSpace, keywords are arranged from left to right along the temporal axis, with node size indicating frequency and the duration of a node’s presence reflecting the continuity of scholarly attention (Liu et al., 2025; Yang et al., 2025). The CNKI and WoS Core Collection timelines both show clear thematic evolution in *Suaeda* research, but the developmental trajectories differ substantially. The domestic literature is more closely associated with regional ecological management and utilization, whereas the international literature shows a stronger tendency toward mechanism-based inquiry and process-oriented explanation.

**Figure 6:**
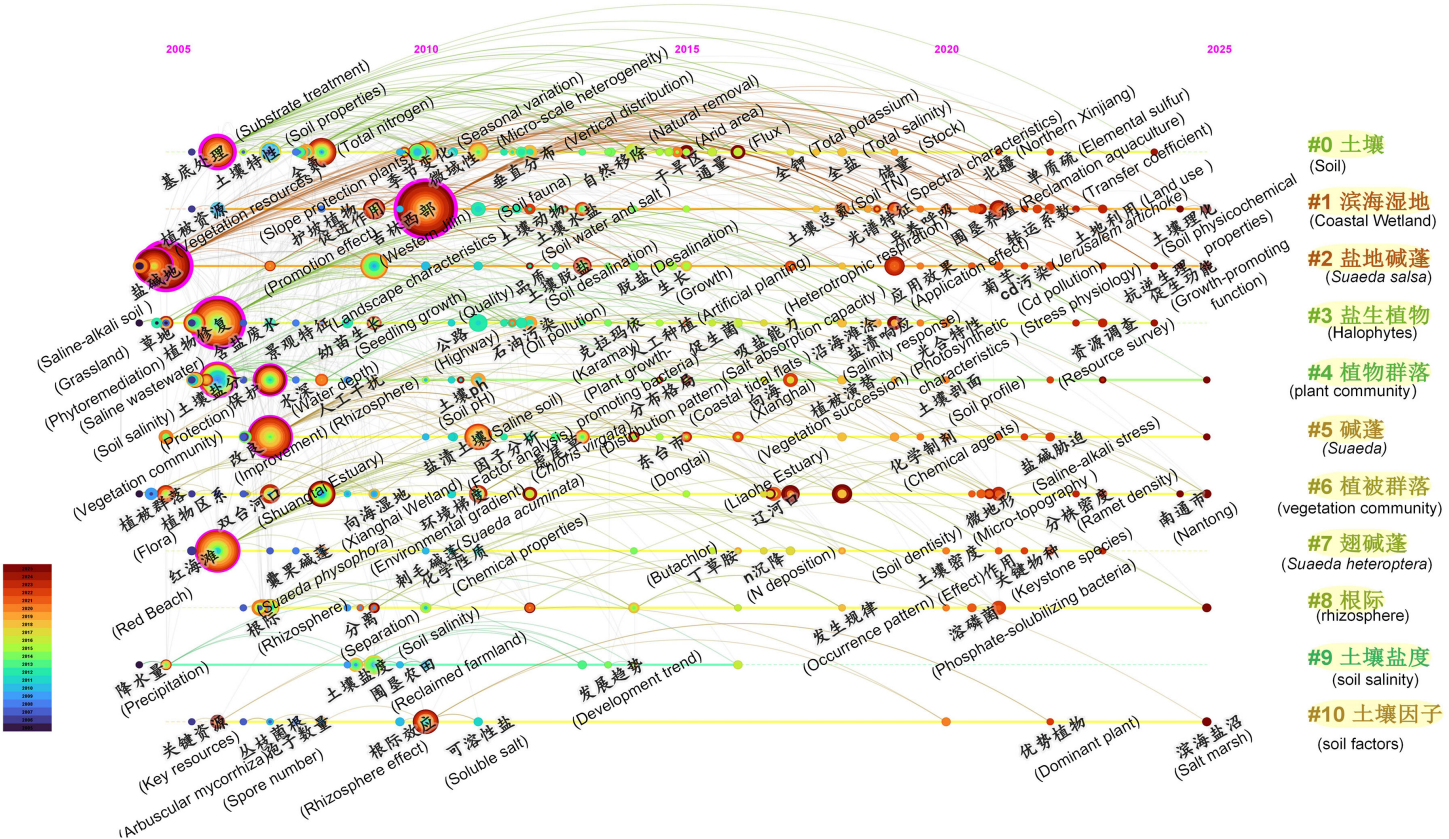
Timeline mapping of keywords from the databases of CNKI (N = 456, E = 974; density = 0.0094; Modularity Q = 0.5764; Weighted Mean Silhouette S = 0.857; Harmonic Mean (Q, S) = 0.6892).

In the CNKI database (**Figure 6**), the timeline analysis covered 2005–2025, generating a network with 456 nodes and 974 links. The domestic frontier developed from habitat and resource investigation to saline-alkali land improvement and ecological restoration, and then toward more diversified functional and technological applications. Persistent keywords such as “saline-alkali land (盐碱地)”, “vegetation resources (植被资源)”, “phytoremediation (植物修复)”, “improvement (改良)” indicate that Chinese *Suaeda* research has long been embedded in practical ecological problems epeated appearance of place-based terms (Muhaxi and Sai’erjiang·Wuermanbieke, 2011; Xiang et al., 2023), including “Western Jilin” (Liu et al., 2024; Sun et al., 2011) and representative coastal wetland regions (Bi et al., 2025; Gong and Li, 2015; Li et al., 2024), further shows that research priorities were strongly shaped by representative saline-alkali and coastal wetland regions. In this sense, frontier evolution in the CNKI dataset remained closely tied to local ecological contexts rather than being driven only by abstract thematic expansion.

**Figure 7:**
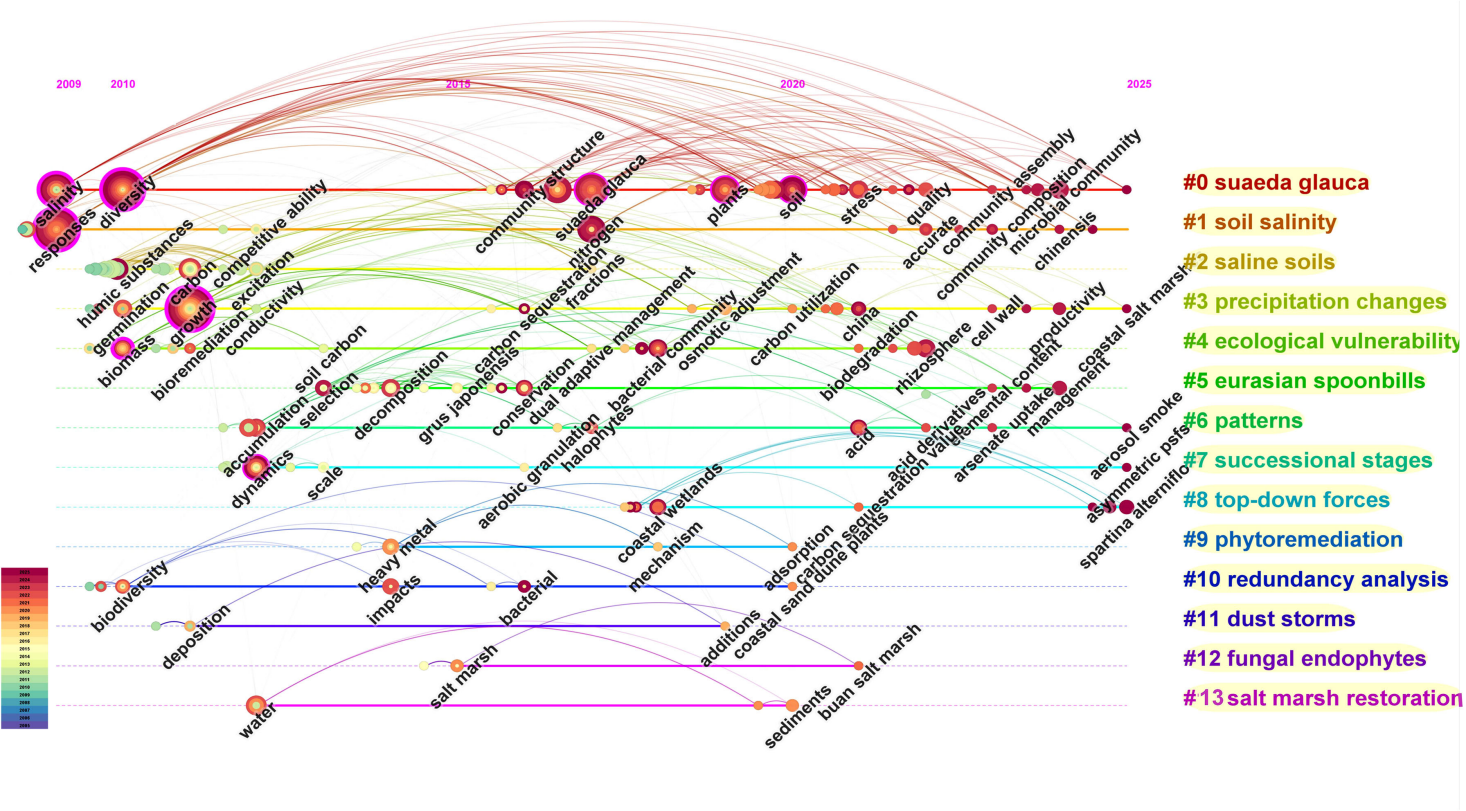
Timeline mapping of keywords from the databases of WoS (N = 343, E = 1239; density = 0.0211; Modularity Q = 0.66; Weighted Mean Silhouette S = 0.8622; Harmonic Mean (Q, S) = 0.7477).

The WoS Core Collection database (**Figure 7**) presents a different pattern. The timeline covered 2010–2025, producing a network with 343 nodes and 1239 links. The international frontier moved from macroecological responses to saline environments toward physiological, molecular, and multi-scale regulatory mechanisms. Compared with the CNKI dataset, the WoS literature shows a stronger orientation toward explanatory depth. Research themes are less anchored in specific regional management settings and more often organized around ecological processes and biological mechanisms. High-frequency keywords such as “salinity”, “response”, “diversity”, “biomass”, “growth”, and “nitrogen” indicate sustained attention to population performance, productivity, nutrient dynamics, and community structure in saline-alkali habitats (Certain et al., 2021; Hwang et al., 2025; Xu et al., 2024b). At this stage, *Suaeda* was examined primarily as a halophytic component of coastal and estuarine ecosystems, with emphasis on its ecological role under salt stress and along environmental gradients (Xia et al., 2022).

A clear shift occurred when the frontier turned toward physiological and molecular mechanisms. Keywords such as “osmotic adjustment”, “antioxidant system”, “heavy metal stress”, “microbial community”, and “rhizosphere interaction” show that the focus moved from external ecological patterns to the internal basis of stress adaptation. Research increasingly addressed how *Suaeda* regulates ion balance, maintains physiological stability, interacts with microorganisms, and responds to complex environmental pressures. In more recent years, this mechanistic orientation has expanded into a more integrated framework. Frontier terms such as “asymmetric PSFs”, “cell wall”, “rhizosphere”, “productivity”, and “arsenate uptake” suggest growing interest in plant-soil feedback, subcellular tolerance mechanisms, rhizosphere-mediated nutrient transformation, and contaminant uptake (Cui et al., 2022; Zhang et al., 2023; Zheng et al., 2026). The WoS timeline therefore does not simply indicate a move toward more microscopic research; it shows the emergence of a more integrative analytical framework linking molecular regulation, microbial processes, ecological interaction, and environmental application.

## 4 Discussion

In recent years, saline-alkali land has gained widespread attention as an important reserve of arable land resources. Previous studies have primarily focused on regulating water and salt dynamics through measures such as drip irrigation and subsurface drainage to achieve sustainable and cost-effective plant production while protecting soil and water resources. However, for moderate to severe saline-alkali land, relying on soil amelioration alone is insufficient. It is necessary to integrate the strategies of “adapting crops to the land” and “adapting land to the crops.” This calls for further research on halophytes such as *Suaeda*. The deepening of this research trend is reflected in the continuously increasing annual number of *Suaeda*-related publications. This delayed but accelerating growth may mirror growing global concerns over soil salinization, coastal wetland degradation, and the ecological functions of halophytes under changing environmental conditions (Zörb et al., 2019). Based on it, we conducted a bibliometric analysis of all Chinese and English-language literature on *Suaeda* published between 2005 and 2025, visualizing and analyzing publication volume, countries, institutions, keywords, and keyword timelines using CiteSpace.

Publication output and temporal trends indicated that despite the substantially higher publication volume and earlier peak observed in the CNKI database, the rapid growth in WoS publications in recent years indicates the increasing internationalization of *Suaeda* research. Core institutions such as the Chinese Academy of Sciences also play an important role in connecting different research teams and facilitating knowledge exchange (**Figure 3**). It suggests that the research on *Suaeda* has gradually expanded from a regionally concentrated applied topic into a broader field of international interest. China’s early start and rapid growth are driven by its vast saline-alkali land area, strong demand for ecological restoration, and supportive policies. The recent rapid growth in international research is driven by studies on salt stress mechanisms, global change, and the rise of functional studies on halophytes. Additionally, the spatial distribution of productive institutions shows a clear correspondence with the ecological distribution and application contexts of *Suaeda*. Many highly productive institutions are located in coastal provinces and municipalities such as Jiangsu, Shandong, and Tianjin, where tidal flats, salt marshes, and estuarine wetlands provide important natural settings for *Suaeda* research. At the same time, institutions in inland saline-alkali regions such as Xinjiang and Inner Mongolia also made substantial contributions, indicating that the research scope of *Suaeda* has expanded from coastal wetland systems to inland saline-alkali ecosystems. This pattern reflects the dual orientation of the field toward both ecological restoration and land-use application. However, the network remains uneven, as some highly productive institutions show relatively limited centrality and weaker integration into broader collaboration frameworks (**Table 1**). This might be due to long-term naming inconsistencies and differences in research directions.

Nomenclatural heterogeneity is evident in the Chinese-language literature, particularly in the inconsistent use of “碱蓬”, *S. glauca*, and *S. salsa*. This inconsistency has also led to long-term cognitive confusion in history. Due to the vague descriptions in the ancient text 《*Jiu Huang Ben Cao*》 (Zhao, 1957), many subsequent medicinal works incorrectly classified *S. salsa* as *S. glauca*. It was not until the 《*Yao Xing Kao*》 from the Qing Dynasty (Zhu., 2008), which recorded that “by autumn, both stems and leaves turn red,” that the uniqueness of *S. salsa* was truly and clearly distinguished. Such nomenclatural inconsistency may affect cross-study comparability, species-level interpretation, and the integration of research findings. Morphologically, *S. glauca* has a “robust” shape and tends to grow upward, whereas *S. salsa* is comparatively “weak” and often spreads horizontally. Moreover, *S. salsa* can modify its morphology (e.g., leaf succulence, stem vascular tissue) in response to different environmental conditions, thereby coping with challenges such as high salinity and waterlogging. Inconsistent naming directly leads to untrustworthy salt tolerance data and non-replicable repair effects. In fact, the academic community confirmed in 2019 that there are 20 species and 1 variety of *Suaeda* plants in China. This might be the reason why the current research cooperation on *Suaeda* is relatively weak. Given that different *Suaeda* species vary in habitat distribution, ecological function, and stress-response strategies, greater taxonomic precision will be important for future studies aiming to synthesize species-specific evidence or develop targeted applications. Thus, the coexistence of multiple species names in the domestic dataset highlights the need for clearer taxonomic standardization in future *Suaeda* research.

On the other hand, keywords provide a condensed representation of the core content and thematic focus of published studies. Keyword network and cluster analysis (**Figures 4**, **5**) reveal complementary patterns between the two datasets. In the CNKI database, high-frequency keywords such as “滨海湿地 (coastal wetlands)”, “盐生植物 (halophytes)”, “土壤 (soil)” reflect the practical orientation of domestic research, which has long been closely linked to coastal wetland management, vegetation reconstruction, and the utilization of salt-tolerant plants under regional ecological conditions (**Table 2**). Typical cases include the Weihai Blue Carbon Industry Chain Project (2014), Panjin Red Beach Wetland Restoration Project (2019), Xinjiang Abi Lake Wetland Suaeda Trial Planting Project (2022), Dongying Hekou District Coastal Zone Ecological Restoration Project (2025). Clusters such as “coastal wetland,” “*Suaeda salsa*,” “halophytes,” and “plant community” are closely connected to practical issues like saline-alkali land improvement, phytoremediation, reclamation, and wetland vegetation management. The frequent occurrence of regional terms (e.g. “Yellow River Estuary (黄河口)”, “Liaohe Estuary (辽河口)”, and “Red Beach (红海滩)”) further shows that domestic studies are deeply rooted in typical Chinese coastal wetlands and driven by local ecological needs. In contrast, the WoS Core Collection database contains clusters such as “precipitation changes”, “ecological vulnerability”, “top-down forces”, and “salt marsh restoration”, which reflect broader concern with global change, ecosystem regulation, and restoration science. These differences also suggest that domestic research has been more strongly linked to regional ecological practice, whereas international research places greater emphasis on general ecological principles and transferable mechanisms. The divergence between application-oriented and mechanism-oriented research stems from differences in resource endowment, management demands, and disciplinary traditions.

In addition, timelines of frontier development reveal different research logics between the two datasets (**Figures 6**, **7**). In the CNKI dataset, early domestic literature was largely descriptive, focusing on distribution patterns, habitat characteristics, vegetation communities, and resource surveys. Research gradually extended beyond coastal salt marshes into inland saline-alkali regions, especially in northwestern and northeastern China (Huang et al., 2026; Xu et al., 2024a), indicating that the field was already moving toward a broader ecological scope. As the field matured, the frontier shifted more clearly toward ecological application. Keywords associated with phytoremediation, artificial planting, saline-alkali land improvement, and rhizosphere processes suggest that *Suaeda* increasingly came to be studied as a functional resource in restoration and environmental management. The shift was not simply from basic research to application. Terms related to rhizosphere soil and stress physiology show that domestic research also began to move toward process-based investigation, particularly in relation to salt tolerance and plant-soil interactions. More recent frontier terms, including those associated with stress resistance physiology (Bu et al., 2024), pollution remediation (Yu et al., 2018), germplasm evaluation (Yan et al., 2023), and spectral or remote sensing applications (Li et al., 2025), suggest that domestic *Suaeda* research is no longer confined to restoration practice in a narrow sense. The plant is increasingly treated as a multifunctional biological resource relevant to ecological assessment, environmental monitoring, and saline-alkali land management (He et al., 2025; Jiang et al., 2025; Qi et al., 2020). The appearance of inland hotspots such as Northern Xinjiang further shows that the domestic frontier continues to expand with changing ecological scenarios and management demands (Huang et al., 2026; Xu et al., 2024a).

In the WoS dataset, high-frequency keywords such as “salinity”, “response”, “diversity”, “biomass”, “growth”, and “nitrogen” indicate sustained attention to population performance, productivity, nutrient dynamics, and community structure in saline-alkali habitats (Certain et al., 2021; Hwang et al., 2025; Xu et al., 2024b). At this stage, *Suaeda* was examined primarily as a halophytic component of coastal and estuarine ecosystems, with emphasis on its ecological role under salt stress and along environmental gradients (Xia et al., 2022). A clear shift occurred when the frontier turned toward physiological and molecular mechanisms. Keywords such as “osmotic adjustment”, “antioxidant system”, “heavy metal stress”, “microbial community”, and “rhizosphere interaction” show that the focus moved from external ecological patterns to the internal basis of stress adaptation (Hao et al., 2020; Ma et al., 2024; Zakery-Asl et al., 2014). Research increasingly addressed how *Suaeda* regulates ion balance, maintains physiological stability, interacts with microorganisms, and responds to complex environmental pressures. In more recent years, this mechanistic orientation has expanded into a more integrated framework. Frontier terms such as “asymmetric PSFs”, “cell wall”, “rhizosphere”, “productivity”, and “arsenate uptake” suggest growing interest in plant-soil feedback, subcellular tolerance mechanisms, rhizosphere-mediated nutrient transformation, and contaminant uptake (Cui et al., 2022; Zhang et al., 2023; Zheng et al., 2026). Thus, the WoS path more often ran from ecological observation to mechanistic explanation and then to broader links with remediation and ecosystem processes.

In summary, domestic research has stronger experience in region-specific restoration and saline-alkali land utilization, while international research offers deeper work on stress physiology, plant–soil interaction, and multi-scale regulation. The contrast is not simply one of topic, but of research logic. Domestic studies more often started from practical environmental problems and then moved toward mechanism, while international studies more often started from ecological and biological questions and later extended toward application. These two lines are beginning to converge. In the CNKI dataset, changes in research focus were driven mainly by ecological management needs, saline-alkali land use, and restoration practice, with mechanistic work added gradually as the field expanded; and in the WoS dataset, the path more often ran from ecological observation to mechanistic explanation and then to broader links with remediation and ecosystem processes. Insufficient international collaboration limits germplasm sharing, omics exchange, and co-development of mechanistic models. Bringing these approaches closer together would help future *Suaeda* research connect restoration practice with physiological adaptation, soil processes, and environmental resilience more effectively.

## 5 Strengths and limitations

To better understand the soil improvement mechanism of *Suaeda* as a halophyte and its application scenarios, this study performed a bibliometric analysis of 1149 publications from the WOS Core Collection and the CNKI database. It systematically examined the overall publication trends, leading countries and institutions, high-frequency keywords, keyword clusters, research frontiers, and future directions in this field, thereby revealing the core research hotspots and development trajectories.

However, several limitations of this study should be acknowledged. First, the literature review was based solely on English-language publications from the WOS Core Collection and Chinese-language publications from the CNKI database, which may have resulted in an incomplete coverage of relevant studies. Second, the bibliometric methods used in this study primarily rely on citation-based indicators, which may not fully represent the actual scientific impact of research outputs. Consequently, some emerging research areas that are potentially significant may be underestimated due to their relatively low citation counts. Third, due to the dynamic nature of database updates, and to ensure comparability between Chinese and English sources, we only included publications from 2005 to 2025. Although the number of studies published before 2005 was fewer than ten, this approach may also have led to the omission of some high-quality studies published more recently.

## 6 Conclusions and outlook

**Figure 8:**
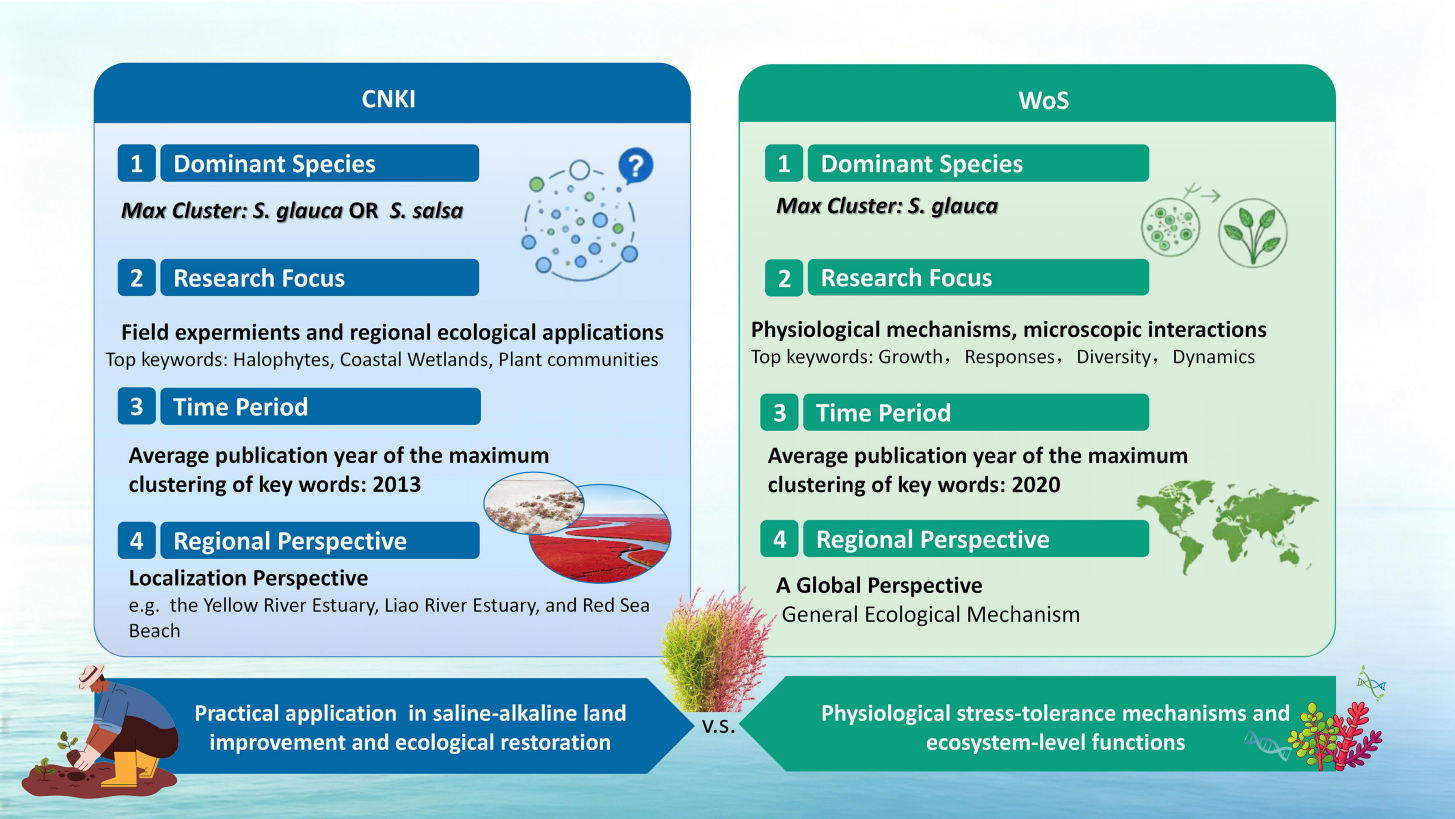
Different development directions of *Suaeda* research in China and internationally.

This study conducted a comparative bibliometric analysis of research on the genus *Suaeda* in the CNKI and Web of Science Core Collection databases from 2005 to 2025. Several conclusions can be drawn (**Figure 8**).

1. The annual number of publications in both databases showed an overall upward trend, although with fluctuations.
2. The overall connectivity of the collaboration network remains relatively limited, suggested that Chinese and international research are complementary (application vs. mechanism), but integration remains weak.
3. Species naming confusion in the Chinese-language literature seriously undermines data reliability.
4. The knowledge structure of the field shows a clear difference between domestic and international research priorities. Domestic studies are more strongly oriented toward applied themes, especially saline-alkali land improvement, ecological restoration, and phytoremediation, whereas international studies focus more on physiological and ecological mechanisms, environmental responses, and ecosystem functions.

Based on the above conclusions, research on the genus *Suaeda* is shifting from regionally grounded and application-oriented exploration toward a more integrated framework combining ecological practice, physiological mechanisms, and multi-scale regulation. Future efforts should focus on the following priorities. First, a global unified standard for *Suaeda* species identification should be established to resolve taxonomic inconsistencies and improve cross-study comparability. Second, stronger integration of restoration applications with salt-tolerance physiology and molecular mechanisms is needed to bridge the gap between fundamental biology and practical saline-alkali land management. Third, Sino-foreign collaborative networks should be built to facilitate sharing of germplasm resources, stress physiology data, and rhizosphere microbiome information, thereby enhancing international cooperation. Fourth, multi-scale salt stress adaptation studies should be conducted across the cellular, individual, community, and ecosystem levels to elucidate the regulatory mechanisms underlying *Suaeda*’s resilience. Such efforts will deepen scientific understanding of *Suaeda* and better support the sustainable utilization and ecological restoration of saline-alkali land.

## Data Availability

The original contributions presented in the study are included in the article/supplementary material. Further inquiries can be directed to the corresponding author.

## References

[B1] Bi J. X. Ma X. J. Liu P. X. Gao R. Zhao T. Yuan X. X. . (2025). Comparative analysis of nutritional composition and medicinal potential between two halophytic species Suaeda salsa and Suaeda heteroptera. Ind. Crops Prod. 237, 122266. doi: 10.1016/j.indcrop.2025.122266 38826717

[B2] Bu X. Li S. Duan Y. Wang Y. Zheng L. (2024). Effects of nitric oxide on stress resistance and feed quality of Suaeda salsa under saline-alkali stress. Acta Pratacult. Sin. 33 (9), 60–69.

[B3] Certain C. Della Patrona L. Gunkel-Grillon P. Léopold A. Soudant P. Le Grand F. (2021). Effect of salinity and nitrogen form in irrigation water on growth, antioxidants and fatty acids profiles in halophytes Salsola australis, Suaeda maritima, and Enchylaena tomentosa for a perspective of biosaline agriculture. Agronomy-Basel 11, 449. doi: 10.3390/agronomy11030449 30654563

[B4] Chen Y. Wu Y. Sun P. Wu D. (2017). Purification of slightly salt-alkaline water bodies by microorganism enhanced combined floating bed. Huanjing. Kexue. 38, 2850–2858. doi: 10.13227/j.hjkx.201610128 29964625

[B5] Chen Y. Zhu J. Wang L. Ning P. Huang W. Zou Z. (2024). Research overview and development of Impatiens L.: a bibliometric analysis, (1987-2023). Horticulturae 10, 1208. doi: 10.3390/horticulturae10111208 30654563

[B6] Cui L. Pan X. Li W. Zhang X. Liu G. Song Y. . (2019). Phragmites australis meets Suaeda salsa on the "red beach": Effects of an ecosystem engineer on salt-marsh litter decomposition. Sci. Tot. Environ. 693, 133477. doi: 10.1016/j.scitotenv.2019.07.283 31362230

[B7] Cui X. Jia B. B. Diao F. W. Li X. Xu J. Zhang Z. C. . (2022). Transcriptomic analysis reveals the molecular mechanisms of arbuscular mycorrhizal fungi and nitrilotriacetic acid on Suaeda salsa tolerance to combined stress of cadmium and salt. Proc. Saf. Environ. Prot. 160, 210–220. doi: 10.1016/j.psep.2022.02.019 38826717

[B8] Geng Y. Jiang X. Bai W. Yan Y. Gao J. (2024). Research progress of tourism marketing over 30 years: Bibliometrics based on CiteSpace. Ecol. Indic. 162, 112059. doi: 10.1016/j.ecolind.2024.112059 38826717

[B9] Gong H. Li S. (2015). Study on plant community succession and diversity gradients in Jiangsu tidal flat reclamation area. Ecol. Sci. 34 (6), 16–21.

[B10] Hao X. Y. Li J. P. Gao S. Q. Tuerxun Z. Chang X. C. Hu W. R. . (2020). SsPsaH, a H subunit of the photosystem I reaction center of Suaeda salsa, confers the capacity of osmotic adjustment in tobacco. Genes Genomics 42, 1455–1465. doi: 10.1007/s13258-020-00970-4 33155109

[B11] He S. Ye H. Zhao Y. Fan R. Zhang Y. Hong M. (2025). Characteristics and ecological function prediction of rhizosphere bacterial communities of different halophytes in saline-alkali land of the Yellow River irrigation area. Acta Ecol. Sin. 45 (5), 2141–2151.

[B12] Hong Y. Kim G. Park Y. Jo H. Nam M. Kim D. . (2023). Suaeda glauca attenuates liver fibrosis in mice by inhibiting TGF β1-Smad2/3 signaling in hepatic stellate cells. Nutrients 15, 3740. doi: 10.3390/nu15173740 37686772 PMC10490352

[B13] Huang J. Shang Y. Chen Y. Xu L. Yang Y. Zhao X. (2025). Analysis of research trends and comprehensive utilization solutions for saline-alkali land. Sustainability 17, 5202. doi: 10.3390/su17115202 30654563

[B14] Huang Y. X. Wang J. Y. Song J. Z. Wang Q. (2026). Phenotypic, pot-experimental, and genomic characterization of Staphylococcus succinus NYN-1, a moderately halophilic bacterium isolated from the rhizosphere of the halophyte Suaeda dendroides in Xinjiang. Microorganisms 14, 680. doi: 10.3390/microorganisms14030680 41900439 PMC13029472

[B15] Hwang J. H. Park J. S. Han Y. S. Yarish C. Kim J. K. (2025). Seasonal variations in biomass, height, photosynthetic efficiency, and carbon and nitrogen contents of Suaeda japonica in Incheon salt marshes (Korea). Front. Plant Sci. 16, 1513624. doi: 10.3389/fpls.2025.1513624 40134615 PMC11933049

[B16] Jiang S. Hu Y. Li D. Jin X. Sun X. (2025). Characteristics of heavy metal mass fractions and pollution assessment in coastal marsh soils after aquaculture withdrawal and wetland restoration. J. Northeast. Forestry. Univ. 53 (10), 100–107.

[B17] Li G. Cai Z. J. Huang S. Z. Song Z. J. Zhang Y. M. Zheng Z. . (2024). Heavy metal accumulation and health risk assessment in S. alterniflora Loisel. and native plant Suaeda salsa (L.) Pall. in Dongtai Tiaozini wetland. Front. Environ. Sci. 12, 1299139. doi: 10.3389/fenvs.2024.1299139

[B18] Li Y. Wang Z. Zhao C. Jia M. Ren C. Mao D. . (2025). Remote sensing-based monitoring and identification mechanisms of the spatiotemporal dynamics of Suaeda salsa in the Liaohe Estuary, China. Remote Sens. For. Natural Resour. 37 (1), 195–203.

[B19] Liu L. Kang F. Xu J. Liu Z. Gong H. He Z. . (2026). Salt barriers produce optimal effects in increasing crop yield and reducing salinity in semi-arid and coastal areas compared to other regions: A meta-analysis. Field Crops Res. 335, 110195. doi: 10.1016/j.fcr.2025.110195 38826717

[B20] Liu L. Liu X. Du C. Fang H. Zhang J. Li W. . (2022). Spring diet and energy intake of whooper swans (Cygnus cygnus) at the Yellow River National Wetland in Baotou, China. PloS One 17, e0264528. doi: 10.1371/journal.pone.0264528 35226691 PMC8884505

[B21] Liu W. Li Y. Jiang H. Wang Y. Chen L. Wang Y. (2024). Comparative analysis of soil microbial composition of four typical plant communities in Momoge National Nature Reserve, Jilin Province. Chin. J. Ecol. 43 (10), 2988–2998.

[B22] Lu X. Zhang X. Liu K. Chen Y. (2024). Review of global studies on remote sensing of Suaeda salsa in the coastal wetlands. J. Suzhou. Univ. Sci. Technol. Nat. Sci. Edition. 41, 40–50.

[B23] Liu Z. Hu B. Zhao Y. Zhang S. Duan X. Liu H. . (2025). Visual analysis of research progress on the impact of cadmium stress on horticultural plants over 25 years. Horticulturae 11, 28. doi: 10.3390/horticulturae11010028 30654563

[B24] Ma L. Li J. Song Y. Li M. Wang X. Guo H. E. (2021). Bibliometric-based analysis and prospect on Suaeda salsa research. Chin. Wild Plant Resour. 40 (2), 57–62.

[B25] Ma J. Z. Xin X. Cao Y. Zhao L. Y. Zhang Z. H. Zhang D. J. . (2024). Root growth characteristics and antioxidant system of Suaeda salsa in response to the short-term nitrogen and phosphorus addition in the Yellow River Delta. Front. Plant Sci. 15, 1410036. doi: 10.3389/fpls.2024.1410036 38911979 PMC11191639

[B26] Muhaxi Sai’erjiang Wuermanbieke (2011). Drip Irrigation Conditions within the Scope of Mixed Deposited Salt Resistant to Salt Grass and the Changing Trend of Salt. Journal of Irrigation and Drainage. 30 (5), 108–110. (in Chinese)

[B27] Ni X. Zhao G. White J. Yao P. Xu K. Sapkota Y. . (2025). Source and degradation of soil organic matter in different vegetations along a salinity gradient in the Yellow River Delta wetland. Catena 248, 108603. doi: 10.1016/j.catena.2024.108603 38826717

[B28] Qi Y. Guan X. He J. Fu G. Cao M. Zhao C. . (2020). Distribution and ecological risk assessment of polycyclic aromatic hydrocarbons in tidal flats in Yellow River Delta, China. Environ. Sci. Technol. 43, 229–236. doi: 10.1016/j.marpolbul.2021.113110 34798430

[B29] Rodriguez-Uribe L. Higbie S. M. Stewart J. M. Wilkins T. Lindemann W. Sengupta-Gopalan C. . (2011). Identification of salt responsive genes using comparative microarray analysis in upland cotton (Gossypium hirsutum L.). Plant Sci. 180, 461–469. doi: 10.1016/j.plantsci.2010.10.009 21421393

[B30] Song Z. Sun Y. Chen P. Jia M. (2022). Assessing the ecosystem health of coastal wetland vegetation (Suaeda salsa) using the pressure state response model, a case of the Liao River Estuary in China. Int. J. Environ. Res. Public Health 19, 546. doi: 10.3390/ijerph19010546 35010806 PMC8744744

[B31] Sun Y. Jin Z. Wei Z. Aiping W. (2005). Current situation and prospect of Suaeda development in China. J. Beijing Technol. Business. Univ. Nat. Sci. Edition. 23 (1), 1–4. 41743750

[B32] Sun Y. Wang Q. Lu X. D. Okane I. Kakishima M. (2011). Endophytic fungi associated with two Suaeda species growing in alkaline soil in China. Mycosphere 2, 239–248. doi: 10.1007/s11427-008-0091-z 18677603

[B33] Tang L. Zhan L. Han Y. Wang Z. Dong L. Zhang Z. (2023). Microbial community assembly and functional profiles along the soil-root continuum of salt-tolerant Suaeda glauca and Suaeda salsa. Front. Plant Sci. 14, 1301117. doi: 10.3389/fpls.2023.1301117 38046600 PMC10691491

[B34] Wang Z. Cai C. Zhou L. Chen L. Lv P. Li J. (2025b). Peat amendments mediated greenhouse gas emissions of saline-alkaline soil depends on halophytes-specific responses. Plant Soil 515, 2171–2186. doi: 10.1007/s11104-025-07713-y 30311153

[B35] Wang Y. Guo T. Tian C. Zhang K. Zhao Z. Mao X. . (2025a). Effects of root growth on galt leaching and soil structure improvement in saline soils: A case study of Suaeda salsa. Agric. Water Manage. 314, 109533. doi: 10.1016/j.agwat.2025.109533 38826717

[B36] Wang Y. Sun Q. Liu J. Wang L. Wu X. Zhao Z. . (2022). Suaeda salsa root-associated microorganisms could effectively improve maize growth and resistance under salt stress. Microbiol. Spectr. 10 (4), 16. doi: 10.1128/spectrum.01349-22 35950864 PMC9430135

[B37] Xia S. P. Song Z. L. Van Zwieten L. Guo L. D. Yu C. X. Wang W. Q. . (2022). Storage, patterns and influencing factors for soil organic carbon in coastal wetlands of China. Global Change Biol. 28, 6065–6085. doi: 10.1111/gcb.16325 35771205

[B38] Xiang L. Wang Y. Chen J. Zhao Y. (2023). Effects of combined stress of salt and heavy metals on germination and growth of Suaeda salsa and regulation measures. Acta Ecologica Sinica. 43, 3307–3318. (in Chinese)

[B39] Xu Q. Liu H. G. Li M. S. Gong P. Li P. F. Xu Y. B. (2024b). Optimizing water and nitrogen management for saline wasteland improvement: A case study on Suaeda salsa. Agric. Water Manage. 301, 108930. doi: 10.1016/j.agwat.2024.108930 38826717

[B40] Xu Q. Liu H. Li M. Li P. Li L. Xu Y. . (2024a). Effects of planting density on the growth and salt transfer of Suaeda salsa L. under drip irrigation conditions. Chin. J. Grassland. 46 (3), 91–99. https://doi.org/1673-5021(2024)46:3<91:Dgtjxz>2.0.Tx;2-8

[B41] Xu Y. Xu Q. Zhang Q. Liu H. Wang G. Gong P. . (2025b). Impact of irrigation water volume and deep vertical rotating tillage depth on the development and feeding quality of Suaeda salsa under drip irrigation. Ind. Crops Prod. 229, 120993. doi: 10.1016/j.indcrop.2025.120993 38826717

[B42] Xu C. Yang T. Wang K. Guo L. Li X. (2023). Knowledge domain and hotspot trends in coal and gas outburst: a scientometric review based on CiteSpace analysis. Environ. Sci. pollut. Res. 30, 29086–29099. doi: 10.1007/s11356-022-23879-9 36401701

[B43] Xu D. Zhang X. Liu J. Zhang Z. Qin C. Zhao Y. . (2025a). Synergistic remediation of cadmium pollution in saline-alkali soil by hydrogel and Suaeda salsa. ACS Appl. Mat. Interfaces. 17, 3911–3923. doi: 10.1021/acsami.4c18057 39762154

[B44] Yan M. Wei X. Cao J. Lan H. (2023). Cloning of basic helix-loop-helix(bHLH) transcription factor gene SabHLH169 in Suaeda aralocaspica and analysis of its resistances to drought stress. Biotechnol. Bull. 39 (11), 328–339. https://doi.org/1002-5464(2023)39:11<328:Yzpsjy>2.0.Tx;2-c

[B45] Yang X. Liu Q. (2024). Research foundation and hotspot analysis of urban road ecology-a bibliometric study based on CiteSpace. Sustainability 16, 5135. doi: 10.3390/su16125135 30654563

[B46] Yang C. Liu Y. Dong Y. Hao S. Liu Y. Qin Y. (2025). Analysis of research hotspots and trends in confined-space work safety based on CiteSpace. Appl. Sci.-Basel. 15, 2452. doi: 10.3390/app15052452 30654563

[B47] Yu Y. Ma M. Xu W. Guo J. Li S. (2018). Study on the remediation of soil contaminated by crude oil using Suaeda salsa in Yellow River Delta. Ecol. Environ. Sci. 27 (10), 1958–1965. https://doi.org/1674-5906(2018)27:10<1958:Jpxfhh>2.0.Tx;2-r

[B48] Yu H. Xi B. Ma W. Li D. He X. (2011). Fluorescence spectroscopic properties of dissolved fulvic acids from salined flavo-aquic soils around Wuliangsuhai in Hetao irrigation district, China. Soil Sci. Soc Am. J. 75, 1385–1393. doi: 10.2136/sssaj2010.0373

[B49] Yu H. Xi B. Su J. Ma W. Wei Z. He X. . (2010). Spectroscopic properties of dissolved fulvic acids: An indicator for soil salinization in arid and semiarid regions in China. Soil Sci. 175, 240–245. doi: 10.1097/SS.0b013e3181e055b4 42241588

[B50] Yue L. Shu-qing Y. Wan-feng Z. Shuai L. (2023). Effect of phosphogypsum and Suaeda salsa on the soil moisture, salt, and bacterial community structure of salinized soil. Huanjing. Kexue. 44, 2325–2337. doi: 10.13227/j.hjkx.202204217 37040981

[B51] Zakery-Asl M. A. Bolandnazar S. Oustan S. (2014). Effect of salinity and nitrogen on growth, sodium, potassium accumulation, and osmotic adjustment of halophyte Suaeda aEgyptiaca (Hasselq.) Zoh. Arch. Agron. Soil Sci. 60, 785–792. doi: 10.1080/03650340.2013.841889 37339054

[B52] Zhang X. Deng Y. Chi G. Lü H. Zhao H. (2025b). Removal of petroleum hydrocarbons from contaminated soils: Analyses of soil enzymes and microbial community evolution during phytoremediation using Suaeda salsa. Pedosphere 35, 591–601. doi: 10.1016/j.pedsph.2024.04.001 38826717

[B53] Zhang L. Kong X. Liu L. Zhang J. Wang Y. Liu H. . (2025a). Phytolith-occluded carbon production of Suaede Salsa promoted by silicon and organic matter. J. Soil Sci. Plant Nutr. 25, 10872–10883. doi: 10.1007/s42729-025-02838-6 30311153

[B54] Zhang L. Xu H. Zhao G. (2007). Salt tolerance of Suaeda Salsa and its soil ameliorating effect on coastal saline soil. Soils. 39 (2), 310–313. doi: 10.13758/j.cnki.tr.2007.02.027

[B55] Zhang S. L. Yin X. T. Arif M. Chen S. S. Ma M. H. Zhu K. . (2023). Strategy matters: Phytoremediation potential of native halophytes is jointly associated with their distinct salt tolerances. J. Cleaner. Prod. 425, 139060. doi: 10.1016/j.jclepro.2023.139060 38826717

[B56] Zhao X. M. (1957). Bencao gangmu shiyi (Beijing: People's Medical Publishing House).

[B57] Zhao H. Zhang R. (2015). Achievement of Nongzheng Quanshu in area of herbalogical textual research. China J. Chin. Materia. Med. 40, 4709–4710. 27141687

[B58] Zheng X. N. Yin Y. H. Yang D. Bi J. J. He W. L. Li S. Y. . (2026). Plant-soil feedback driven by root-associated fungal communities accelerates the secondary succession of bare saline-alkaline grassland patches. Plant Soil 519, 435–450. doi: 10.1007/s11104-025-08132-9 30311153

[B59] Zhu S. (2008). Jiuhuang bencao jiaozhu. Ed. Ni G. J. (Beijing: China Agriculture Press).

[B60] Zhu W. Xiao Y. Xie L. (2025). Visualization analysis of poisoning-related research based on CiteSpace. Front. Public Health 13, 1592916. doi: 10.3389/fpubh.2025.1592916 40401055 PMC12092380

[B61] Zörb C. Geilfus C. Dietz K. (2019). Salinity and crop yield. Plant Biol. 21, 31–38. doi: 10.1111/plb.12884 30059606

